# Competing Risks and Their Impact on Treatment Efficacy Assessment in Fractionated Stereotactic Radiotherapy for Brain Metastases: A Retrospective Study

**DOI:** 10.1002/hsr2.70447

**Published:** 2025-02-10

**Authors:** Isabella Gruber, Oliver Koelbl

**Affiliations:** ^1^ Department of Radiation Oncology University Hospital Regensburg Regensburg Bavaria Germany

**Keywords:** brain metastases, competing risks, cumulative incidence function, fractionated stereotactic radiotherapy, Kaplan‐Meier, local failure, premature deaths

## Abstract

**Background and Aims:**

The Kaplan‐Meier (KM) method and competing risk analysis are two statistical approaches for analyzing time‐to‐event data. These methods differ in their treatment of competing events, such as deaths occurring before the event of interest, which can impact the interpretation of treatment efficacy in oncology.

**Methods:**

This retrospective study included 73 patients who underwent fractionated stereotactic radiotherapy (six fractions of 5 Gy) for brain metastases at the University Hospital Regensburg between January 2017 and December 2021. The events of interest were the cumulative incidences of local failure within the planning target volume and the development of new brain metastases. Premature deaths occurring before the events of interest were treated as competing events. The complement of the KM method (1‐KM), which censors patients who die prematurely, was compared to the cumulative incidence function (CIF), which accounts for the fact that patients who die without experiencing the event of interest are no longer at risk for that event.

**Results:**

The median follow‐up was 56 months. The most common cancer types were non‐small cell lung cancer (*n *= 26, 35.6%) and malignant melanoma (*n *= 25, 34.2%). Using CIF, the cumulative incidences of local failure and new brain metastases were 27% and 55% at 24 months, respectively. The 1‐KM method overestimated the cumulative incidence of local failure by 9% at 24 months and 14% at 36 months, and new brain metastases by 13% at both 24 and 36 months. Survival curves for 1‐KM and CIF showed simultaneous increases for each event, with 1‐KM consistently higher, reflecting differing approaches to competing risks.

**Conclusion:**

This study highlights the impact of statistical method selection on clinical data interpretation and underscores the bias inherent in studies that fail to account for competing risks. Recognizing these differences is crucial for accurately assessing treatment outcomes.

## 1 Introduction

Accurate analysis of time‐to‐event data is essential for evaluating treatment efficacy in clinical oncology. The Kaplan–Meier method is widely employed in this context due to its simplicity and versatility. However, its limitations become evident in the presence of competing risks ‐ events that preclude the occurrence of the primary event of interest [1, 2, 3]. In conventional Kaplan‐Meier analysis, patients who experience a competing event (e.g., death before reaching the study endpoint) are censored, which assumes they remain at risk for the event of interest (e.g. local relapse of cancer). This assumption leads to an overestimation of the probability of the event of interest, as competing events are not accounted for. As a result, the Kaplan–Meier method may produce inaccurate estimates when competing risks are substantial [1, 2, 4].

Competing risk analyses, particularly the cumulative incidence function (CIF), offer a robust alternative by recognizing that patients who experience competing events are no longer at risk for the event of interest [1, 2, 4]. This distinction is particularly relevant in studies involving frail or elderly populations, where premature deaths are common and can significantly influence the interpretation of clinical endpoints [4]. In the survival analysis of local failure of brain metastases following fractionated stereotactic radiotherapy (FSRT), death prevents the observation of local failure and is thus considered a competing event. Despite the advantages of the CIF, competing risk analyses remain underutilized in clinical oncology, with the Kaplan‐Meier method often applied even when competing risks are present [5].

This retrospective study addresses this gap by comparing the Kaplan‐Meier method (via its complement, 1‐KM) with the CIF in the context of FSRT for brain metastases. This analysis focuses on two clinically relevant endpoints: local failure within the planning target volume (PTV) and the development of new brain metastases outside the PTV. By illustrating the preparation and visualization of time‐to‐event data using both the complement of the Kaplan‐Meier method (1‐KM) and the CIF, this study highlights the critical discrepancies between the two methods and underscores the importance of selecting appropriate statistical approaches to ensure accurate interpretation of treatment outcomes in the presence of competing events.

## 2 Methods

### 2.1 Data Collection

We previously reported outcomes of 73 patients with brain metastases treated with FSRT at the University Hospital Regensburg between January 2017 and December 2021, analyzed using Kaplan–Meier methods [6]. Eligibility criteria included patients with brain metastases of solid cancers undergoing their first FSRT with six fractions of 5 Gy. Patients with a prior history of radiotherapy for brain metastases, including whole‐brain radiotherapy, stereotactic radiosurgery, or FSRT, were excluded. Clinical data were extracted from the medical charts of the University Hospital Regensburg. The variables included patient age, sex, diagnosis, Karnofsky performance score, and recursive partitioning analysis (RPA). The gross tumor volume (GTV) and PTV were used to describe brain metastases.

In this retrospective study, we updated the results with data available as of April 2024 and analyzed the cumulative incidences of local failure within the PTV and new brain metastases outside the PTV, treating premature deaths before reaching the study endpoint as a competing risk for each endpoint. This analysis utilized the CIF, a key tool in competing risk methodology, and compared these findings with standard KM analyses. To reduce confounding factors in a per‐lesion analysis, the study included one brain metastasis per patient. For patients with more than one brain metastasis treated with FSRT, the first treated metastasis was selected.

### 2.2 Fractionated Stereotactic Radiotherapy and Response Assessment

Details of the radiotherapy technique have been previously published [6]. Patients were immobilized using a stereotactic mask system. Diagnostic contrast‐enhanced T1‐weighted magnetic resonance imaging was fused with computed tomography scans to aid in treatment planning. The GTV was defined as the visible volume of brain metastases identified on magnetic resonance imaging. The PTV was generated by adding a uniform isotropic margin of 2–3 mm around the GTV. All patients underwent FSRT, receiving six fractions of 5 Gy each, for a total dose of 30 Gy. Radiotherapy sessions were typically conducted three times per week. Patients received coplanar and noncoplanar 6 megavoltage photon beams using a linear accelerator of type Elekta Synergy™ or SynergyS™ (Elekta Ltd, Crawley, UK).

Patients underwent follow‐up with gadolinium‐enhanced magnetic resonance imaging (MRI) at 6–8 weeks post‐FSRT and subsequently every 3 months. Local failure of the irradiated brain metastases was defined as at least a 20% increase in the sum of the longest diameter of the metastasis [7], accompanied by an increase of ≥5 mm [6]. Local failure and brain radiation necrosis were retrospectively differentiated based on follow‐up imaging or histological findings in cases where surgical resection was performed [6].

### 2.3 Definitions and Statistical Methods

To illustrate the differences between the Kaplan–Meier (KM) method (via its complement, 1‐KM) and competing risk methodology using the CIF, this study analyzed the clinical endpoints: local failure of irradiated brain metastases within the PTV and the development of new brain metastases outside the PTV.

For the 1‐KM analyses, each patient was assigned a binary code: state 1 for the event of interest and state 0 for all other events. The times from the last day of FSRT to the event of interest (e.g., local failure of the irradiated brain metastasis) was recorded. For patients who did not experience the event of interest, the 1‐KM method recorded the time from FSRT to either the last day of follow‐up while alive and event‐free or the day of death without prior occurrence of the event of interest. The 1‐KM method focused solely on the event of interest (state 1). This approach did not differentiate between patients who were event‐free at the last follow‐up and those who died before reaching the study endpoint, treating both scenarios as censored observations [8]. Importantly, censoring assumes that the event of interest would have been observed in all patients if the study duration were extended or if no patients had dropped out. However, this assumption ‐ that patients who died before the event of interest occurred have the same future risk for the event of interest as surviving patients ‐ artificially inflates the estimated cumulative incidences [8].

**Figure 1:**
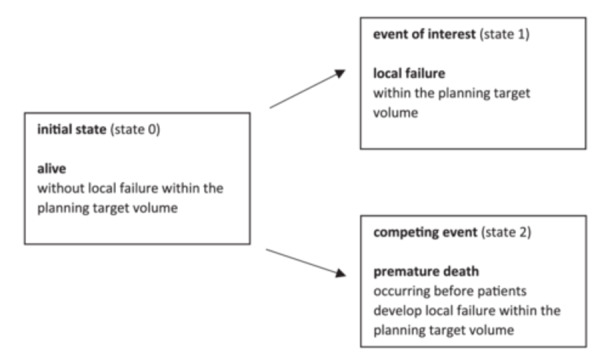
Potential outcomes of the clinical endpoint of local failure within the planning target volume following fractionated stereotactic radiotherapy of brain metastases using competing risk methods.

**Figure 2:**
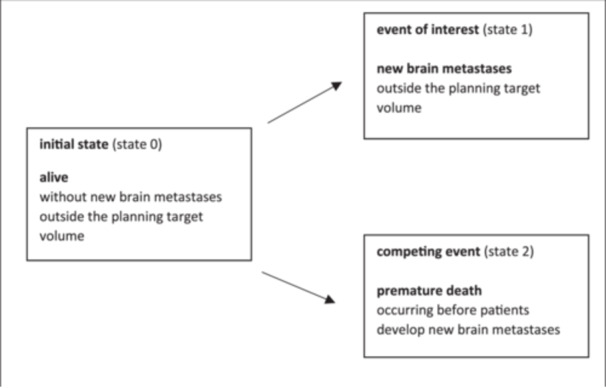
Potential outcomes of the clinical endpoint of new brain metastases outside the planning target volume following fractionated stereotactic radiotherapy of brain metastases using competing risk methods.

In contrast, the CIF describes the probability of experiencing the event of interest before the occurrence of a competing event. The CIF accounts for the fact that deaths remove patients from the risk set for the event of interest, as individuals who have died can no longer be at risk for the event. For the clinical endpoints of local failure within the PTV (Figure 1) and new brain metastases outside the PTV (Figure 2), the CIF classified patients into one of three mutually exclusive states.

Patients were observed from the last day of FSRT until they experienced one of the following: the event of interest (e.g., local failure of the irradiated brain metastasis, state 1), the competing event (death occurring before experiencing local failure, state 2), or the last day of follow‐up without experiencing either the event of interest or the competing event (still at risk, state 0). Since these three states were mutually exclusive, the probabilities of being in any of the three states at any given time summed to 100% [2].

The overestimation of the cumulative incidences of local failure within the PTV or new brain metastases outside the PTV was quantified as the difference between the values obtained using the 1‐KM method and the CIF.

### 2.4 Statistical Analysis

Categorical variables were presented as absolute and relative frequencies, and continuous variables as median and interquartile range (IQR). Median follow‐up time was estimated using the reverse Kaplan‐Meier method. The median survival was determined using the Kaplan‐Meier method. The present study utilized the CIF to analyze the cumulative incidences of local failure and new brain metastases, accounting for the competing risk of death occurring before the events of interest. Hazard ratio (HR) and 95% confidence interval (95% CI) are presented as effect estimate. Statistical analysis was performed using R, version 4.3.2 (R Core Team. R: A language for statistical computing. 2014. The R Foundation for Statistical Computing, Vienna, Austria), using the “tidycmprsk”, “prodlim”, “riskRegression” and “survival” packages.

### 2.5 Ethics Approval

This study was performed in line with the principles of the Declaration of Helsinki. Approval was granted by the Ethics Committee of the University Regensburg (ethics approval number: 22‐2868‐104, date: March 24, 2022). Informed consent was obtained from all individual participants included in the study.

## 3 Results

### 3.1 Patient Characteristics

A total of 91 patients received FSRT between January 2017 and December 2021. Eighteen patients with a prior history of radiotherapy for brain metastases were excluded. A total of 73 patients with 73 brain metastases were analyzed, comprising 40 men (54.8%) and 33 women (45.2%). The median follow‐up duration was 56.0 months (IQR, 34.0‐66.4). Table 1 shows patient characteristics. The median patient age at the time of FSRT was 61 years (IQR, 51‐67). The most common cancer types were non‐small cell lung cancer (NSCLC, *n *= 26, 35.6%) and malignant melanoma (*n *= 25, 34.2%). The median GTV of the irradiated brain metastases was 1.4 cm³ (IQR, 0.5–3.9), translating into a median PTV of 4.1 cm³ (IQR, 2.4–11.8). Most brain metastases were located supratentorially (*n *= 54, 74.0%), while the remainder were infratentorial (*n *= 19, 26.0%). The median overall survival was 13.7 months (IQR, 6.6–37.6).

**Table 1:** Patient and brain metastases characteristics (*n *= 73).

Characteristics	Value
**patient age**, years (IQR)	61 (51–67)
**sex**, *n* (%)	
female	33 (45.2%)
male	40 (54.8%)
**primary cancer**, *n* (%)	
malignant melanoma	25 (34.2%)
non‐small cell lung cancer (NSCLC) adenocarcinoma	19 (26.0%)
NSCLC non‐adenocarcinoma	7 (9.6%)
breast cancer	6 (8.2%)
gastrointestinal carcinoma	5 (6.8%)
renal cell carcinoma	1 (1.4%)
other	10 (13.7%)
**Karnofsky performance status**, median (IQR)	70 (60–90)
**Recursive partitioning analysis** (RPA) score, median (IQR)	2.0 (2.0–2.5)
**extracranial metastases**, *n* (%)	
present	48 (65.8%)
absent	25 (34.2%)
**systemic therapy** ^a^ 3 months before or after FSRT, n (%)	66 (90.4%)

Abbreviation: FSRT, fractionated stereotactic radiotherapy; IQR, interquartile range.

^a^ chemotherapy, immunotherapy, targeted therapy or antihormonal therapy

### 3.2 Local Failure of Irradiated Brain Metastases after Fractionated Stereotactic Radiotherapy

Among all patients, 22 patients (30.1%) developed local failure of the irradiated brain metastases within the PTV (state 1), 38 (52.1%) experienced the competing risk of death without prior local failure (state 2, death before reaching the study endpoint), and 13 (17.8%) remained alive without developing local failure (state 0). Table 2 presents the cumulative incidence rates of local failure within the PTV and the competing risk of death before experiencing local failure, using the complement of the Kaplan‐Meier method (1‐KM) and the CIF.

**Table 2:** Cumulative incidence rates of local failure within the planning target volume and death before experiencing local failure. A competing risk analysis using the complement of the Kaplan–Meier method and cumulative incidence function.

Cumulative incidences with 95% confidence intervals at specific time points	Kaplan‐Meier 1‐KM	Cumulative incidence function CIF
**local failure** ^a^		
12 months	28% (95% CI: 15–39)	23% (95% CI: 14–34)
24 months	36% (95% CI: 21–49)	27% (95% CI: 18–38)
36 months	44% (95% CI: 26–57)	30% (95% CI: 20–41)
**competing risk of death before experiencing local failure** ^a^		
12 months	45% (95% CI: 30–56)	37% (95% CI: 26–48)
24 months	57% (95% CI: 42–68)	45% (95% CI: 33–56)
36 months	65% (95% CI: 48–76)	50% (95% CI: 38–61)

^a^ *local failure*, local failure within the planning target volume

**Figure 3:**
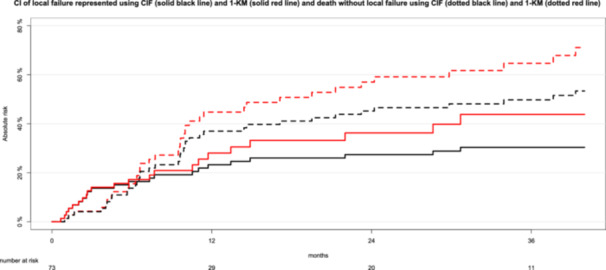
Cumulative incidences (CI) of local failure within the planning target volume (PTV) and cumulative incidences of death before local failure (competing risk) represented using the cumulative incidence function (CIF) and the complement of the Kaplan–Meier method (1‐KM). Black curves depict cumulative incidences calculated using CIF, while red curves represent those derived from the 1‐KM method. Solid lines indicate the cumulative incidences of local failure, whereas dotted lines illustrate the cumulative incidences of the competing risk of death (without prior local failure).

Figure 3 illustrates the cumulative incidences of local failure within the PTV and the competing risk of death before experiencing local failure, comparing results from the complement of the Kaplan‐Meier method (1‐KM) and the CIF.

Using the 1‐KM method (Figure 3, red lines), the cumulative incidence of local failure within the PTV was 36% at 24 months, while the cumulative incidence of the competing event (death without prior local failure) was 57% at the same time point. However, the 1‐KM method assumes that patients who die before developing local failure (the competing event) remain at risk for local failure, leading to an overestimation of the cumulative incidences of local failure. For instance, at 36 months, the cumulative incidences of local failure (44%) and death without prior local failure (65%) sum to an impossible value of 109%. This overestimation occurs because the KM analysis treats patients who die before local failure as if they remain at risk for it.

For comparison, the black curves in Figure 3 show the cumulative incidences of both endpoints using the CIF method. The CIF‐curves reveal that, at 24 months, 27% of patients experienced local failure of the irradiated brain metastasis, 45% experienced the competing event (death without prior local failure), and 28% remained alive without any event. Both the 1‐KM and CIF curves for local failure increase at the same time points when local failure within the PTV occurs, as the event times are identical. However, the magnitude of the increase is more pronounced with the 1‐KM method compared to the CIF. This is because the 1‐KM method includes patients who experienced the competing event (death without prior local failure) in the risk set for local failure, resulting in overestimation. The overestimation of cumulative incidences of local failure by the 1‐KM method was 5% at 12 months, 9% at 24 months, and 14% at 36 months. The discrepancies between the two methods are even more pronounced for the competing event of death without prior local failure, with incidences of 57% for the 1‐KM method versus 45% for the CIF at 24 months.

### 3.3 New Brain Metastases Outside the PTV after Fractionated Stereotactic Radiotherapy

Forty patients (54.8%) developed new brain metastases outside the PTV (event of interest, state 1), 23 patients (31.5%) experienced the competing risk of death without developing new brain metastases (death before reaching the study endpoint, state 2), and 10 patients (13.7%) remained alive without developing new brain metastases (state 0). Table 3 presents the cumulative incidence rates of new brain metastases outside the PTV and death before the development of new brain metastases in a competing risk setting, calculated using the complement of the Kaplan‐Meier method (1‐KM) and the CIF.

**Table 3:** Cumulative incidence rates of new brain metastases outside the planning target volume and death before the development of new brain metastases. A competing risk analysis using the complement of the Kaplan‐Meier method and cumulative incidence function.

Cumulative incidences with 95% confidence intervals at specific time points	Kaplan‐Meier 1‐KM	Cumulative incidence function CIF
**new brain metastases** a		
12 months	53% (95% CI: 39–64)	47% (95% CI: 35–58)
24 months	68% (95% CI: 52–79)	55% (95% CI: 43–66)
36 months	68% (95% CI: 52–79)	55% (95% CI: 43–66)
**competing risk of death before the development of new brain metastases** a		
12 months	39% (95% CI: 22–52)	25% (95% CI: 15–35)
24 months	48% (95% CI: 30–62)	29% (95% CI: 19–40)
36 months	53% (95% CI: 32–68)	30% (95% CI: 20–41)

^a^ *new brain metastases*, outside of the planning target volume

**Figure 4:**
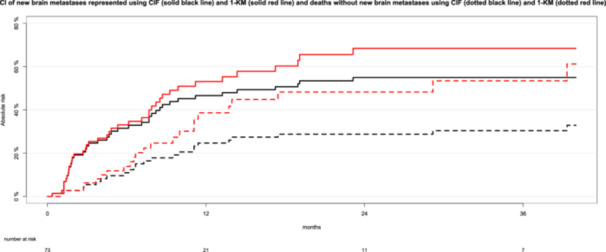
Cumulative incidences (CI) of new brain metastases outside the planning target volume and the competing risk of death before the development of new brain metastases represented using the cumulative incidence function (CIF) and the complement of the Kaplan‐Meier method (1‐KM). Black curves depict the cumulative incidences calculated using CIF, while red curves represent those derived from the 1‐KM method. Solid lines indicate the cumulative incidences of new brain metastases outside the planning target volume (event of interest), whereas dotted lines illustrate the cumulative incidences of the competing risk of death (occurring before the development of new brain metastases).

Cumulative incidence curves for new brain metastases outside the PTV (event of interest) and deaths before the development of new brain metastases (competing risk), estimated using the complement of the Kaplan‐Meier method (1‐KM) and the CIF are shown in Figure 4.

Using the complement of the Kaplan‐Meier method (Figure 4, red lines), the cumulative incidences of new brain metastases outside the PTV was 68% at 24 months. The cumulative incidences of deaths before the development of new brain metastases (competing risk) was 48% at 24 months, using 1‐KM. The curves (black lines) generated using the CIF illustrate that, at the 24 months after FSRT, 55% of patients developed new brain metastases outside the PTV, and 29% died without experiencing new brain metastases (competing event). The remaining 16% were alive without any events.

The curves generated by the complement of the Kaplan–Meier method (1‐KM) and the CIF increase simultaneously when new brain metastases occur. However, the increase is systematically higher with the 1‐KM method compared to the CIF method (e.g., new brain metastases at 24 months: 68% vs. 55%). Failure to account for premature deaths resulted in an overestimation of the cumulative incidence by 13% at 24 months when using the 1‐KM method. The overestimation was even more pronounced for the competing event between the two methods (deaths without prior new brain metastases at 24 months: 29% vs. 48%).

## 4 Discussion

The advanced age and frail status of cancer patients undergoing radiotherapy necessitate statistical methods that account for the probability of death before reaching the study endpoint [4]. Despite the presence of premature deaths in clinical studies, competing risk analyses are rarely employed in clinical trials of Radiation Oncology. Most articles addressing competing risks are published in journals specializing in epidemiology [2, 9, 10] or statistics [11]. Therefore, this study compared competing risk methodology with Kaplan‐Meier methods using data from 73 patients treated with FSRT for brain metastases. Although the times to both endpoints of local failure and new brain metastases were identical, differences in the coding of the status variable between the two methods led to differing cumulative incidences.

Premature deaths occurring before the events of interest were treated as competing risks for both study endpoints, using the CIF, as these deaths precluded the occurrence of the events of interest. However, the complement of the Kaplan–Meier method (1‐KM) did not account for the fact that patients who died were no longer at risk of developing the event of interest. As a result, the 1‐KM method overestimated the incidence of these events compared to estimates derived from the CIF. Our findings demonstrate that the 1‐KM method resulted in a 9% overestimation of local failure incidence at 24 months. Thus, the probability of local failure is more accurately estimated using the CIF than the 1‐KM method. Similarly, neglecting premature deaths before the occurrence of new brain metastases led to an overestimation of the cumulative incidence of new brain metastases by 6% at 12 months and 13% at 24 months. When comparing study results, the statistical method used (CIF vs. KM) should always be considered.

Literature suggests that the proportion of patients experiencing the event of interest relative to those experiencing the competing event determines the magnitude of overestimation. Competing risk analyses are particularly relevant when the proportion of competing events equals or exceeds the proportion of patients experiencing the event of interest or when the percentage of competing events exceeds 10% [1, 2]. Therefore, it is crucial to report the cumulative incidence of deaths without the events of interest alongside the incidence of the event of interest (e.g., local failure), as these events are mutually exclusive. The literature strongly recommends presenting separate CIF‐curves for each event to ensure a comprehensive analysis, as demonstrated in the present study [9]. Different degrees of censoring exist in KM analyses in the context of competing risks [8]. However, many papers fail to specify the censoring methods used, despite the availability of guiding literature [8]. This study provided details about the timing and types of censoring in KM analyses and contrasted these with the procedures used in CIF. A potential reason for the widespread use of KM methods in survival data analysis is their familiarity among researchers, whereas CIF methods may appear more complex and require advanced statistical software [11].

The impact of competing events on results cannot be fully understood unless competing risk analyses are performed. Van Walraven et al. [5] reviewed 100 studies published in medical journals and found that 46% did not account for competing risks despite their presence. Their review demonstrated a median relative increase in risk estimates of 5.7% (IQR, 2.2–12.6) when using KM methods compared to the CIF, which aligns with our findings. Notably, more than one‐third of the studies showed overestimations of risk exceeding 10% [5]. Such overestimations have clinical implications, as they can influence decision‐making based on flawed results.

Studies analyzing secondary malignancies or late toxicity after radiotherapy are particularly susceptible to competing risks, as patients who die of other causes cannot subsequently develop secondary cancer or late toxicity [4]. Medical literature readers and reviewers should be aware of the potential biases introduced by KM analyses in the presence of competing risks [5].

The median age of patients undergoing FSRT for brain metastases in this study was relatively young (61 years). Discrepancies between the 1‐KM method and CIF may become more pronounced in studies involving older patients, where premature deaths are more frequent. Additional limitations of this study include its focus on only two endpoints and the absence of multivariate analysis. It is important to note that studies using all‐cause mortality as the event of interest are not affected by competing risks, as the 1‐KM method accurately estimates incidences in such cases. Moreover, in studies with a low frequency of competing events, estimates from the 1‐KM method and the CIF tend to be similar [4]. Although this study is not intended to serve as a comprehensive statistical tutorial, it emphasizes the fundamentals of competing risk analyses, with a focus on two clinically relevant endpoints in oncology research.

## 5 Conclusion

These results highlight the importance of recognizing the unique characteristics of clinical studies when competing events are present but overlooked. Death before reaching the study endpoint constitutes a significant competing risk for both local failure of irradiated brain metastases and the development of new brain metastases, as deceased patients can no longer experience these events. Censoring patients who die before reaching the study endpoint, as is done in the Kaplan–Meier method, results in an overestimation of the events of interest. Consequently, studies that rely on KM methods in the presence of competing risks are prone to overestimating their results. For endpoints such as local relapse and new brain metastases, the CIF is a more appropriate statistical method, as it accounts for the fact that patients who die prematurely are no longer at risk of developing these events. Although competing risk analyses are not novel in the literature, they remain underutilized in Radiation Oncology. The considerations raised in this study are particularly relevant for research involving older patients, who are at high risk of death. This study underscores the pitfalls of neglecting competing risks in survival data, and provides valuable insights for healthcare professionals conducting or interpreting clinical studies in these contexts.

## Author Contributions

**Isabella Gruber:** Conceptualization, Writing–original draft, Methodology, Visualization, Writing–review and editing, Formal analysis, Project administration, Data curation, Investigation. **Oliver Koelbl:** Writing–review and editing, Supervision, Conceptualization.

## Conflicts of Interest

The authors declare no conflicts of interest.

## Transparency Statement

The lead author Isabella Gruber affirms that this manuscript is an honest, accurate, and transparent account of the study being reported; that no important aspects of the study have been omitted; and that any discrepancies from the study as planned (and, if relevant, registered) have been explained.

## Data Availability

The data that support the findings of this study are available from the corresponding author, Isabella Gruber, upon request. The data are not publicly available due to privacy or ethical restrictions.
